# The Mechanism of Resistance of EUROPEAN Plum to *Plum pox virus* Mediated by Hypersensitive Response Is Linked to VIRAL NIa and Its Protease Activity

**DOI:** 10.3390/plants12081609

**Published:** 2023-04-10

**Authors:** Bernardo Rodamilans, Johannes Hadersdorfer, Zita Berki, Beatriz García, Michael Neumüller, Juan Antonio García

**Affiliations:** 1 Centro Nacional de Biotecnología (CNB-CSIC), Campus Universidad Autónoma de Madrid, Darwin 3, 28049 Madrid, Spain; 2 School of Life Sciences Weihenstephan, Technical University of Munich, Dürnast 2, D-85354 Freising, Germany; 3 Bavarian Centre of Pomology and Fruit Breeding, Am Süßbach 1, D-85399 Hallbergmoos, Germany

**Keywords:** *Plum pox virus*, hypersensitive response, resistance, potyvirus, *Prunus*, plum

## Abstract

*Plum pox virus* (PPV) infects *Prunus* trees across the globe, causing the serious Sharka disease. Breeding programs in the past 20 years have been successful, generating plum varieties hypersensitive to PPV that show resistance in the field. Recently, a single tree displaying typical PPV symptoms was detected in an orchard of resistant plums. The tree was eradicated, and infected material was propagated under controlled conditions to study the new PPV isolate. Performing overlapping PCR analysis, the viral sequence was reconstructed, cloned and tested for infectivity in different ‘Jojo’-based resistant plums. The results confirmed that the isolate, named PPV-D ‘Herrenberg’ (PPVD-H), was able to infect all these varieties. Analyses of chimeras between PPVD-H and a PPV-D standard isolate (PPVD) revealed that the NIa region of PPD-H, carrying three amino acid changes, was enough to break the resistance of these plums. Experiments with single and double mutants showed that all changes were essential to preserve the escaping phenotype. Additionally, one of the changes at the VPg-NIapro junction suggested the involvement of controlled endopeptidase cleavage in the viral response. Transient expression experiments in *Nicotiana benthamiana* confirmed that NIa cleavage in PPVD-H was reduced, compared to PPVD, linking the observed behavior to an NIa cleavage modulation.

## 1. Introduction

Sharka is one of the most devastating diseases of stone fruit trees in the world [1,2,3,4]. First discovered in plum (*Prunus domestica*) [5], this disease affects, amongst other plants, many *Prunus* species such as peach (*P. persica*) and apricot (*P. armeniaca*), and it is estimated that millions of cultivated fruit trees have been infected only in Europe with losses over the past 50 years of more than EUR 10 billion [6,7]. The virus responsible for this disease is *Plum pox virus* (PPV), a positive single-stranded RNA virus of the family *Potyviridae*, one of the largest families of plant viruses [8,9]. Its genome, of approximately 10 Kb, is expressed as one main ORF and two secondary ORFs produced after polymerase slippage [10,11,12,13]. All genomic products of potyviruses are translated as polyproteins that are processed by viral endopeptidases [14]. PPV encodes two proteases at the 5′ end of the genome, the serine protease P1 and the cysteine protease HCPro [4]. They both act in cis for self-release [15,16]. The rest of the protein products are processed by the 3C-like endopeptidase NIapro, a chymotrypsin-like protease present in all viruses of the family that is able to cut in cis and in trans [17]. NIapro is produced as a larger product termed NIa, consisting of VPg and NIapro. Cleavage of the polyprotein by NIapro is tightly regulated and involves the presence of several intermediate uncleaved products that could be relevant at different times of infection [18]. NIapro cleavage has also been related to host adaptation in the case of PPV-SwC [19]. In addition, NIapro has been described as a pathogenicity determinant in Pepper mottle virus [20] and Potato virus Y (PVY) in tobacco [21], and it has been characterized as the elicitor of the *Ry*-mediated resistance in the case of PVY–potato plants interaction [22,23].

PPV is transmitted by aphids in a non-persistent manner [24,25,26], and up to date nine strains of the virus have been identified, with D, M and Rec being the most prevalent [3,27]. Isolates of the PPV-D strain infect, among other species, European plum and cause strong chlorosis in the leaves and deformation in fruits, originating epidermal necrotic tissue of undesirable flavor and sometimes, premature drop [28]. Extensive efforts have been dedicated in the past 30 years towards breeding varieties that could be resistant to PPV in the field. ‘Jojo’, a plum variety derived from a progeny ‘Ortenauer’ × ‘Stanley’, displays strong hypersensitive response (HR) upon artificial PPV infection by grafting [29,30,31,32]. It displays necrosis in leaves and bark and death of new sprouts when tested experimentally with PPV-D, -M, -Rec, -EA and -W strains. On the other hand, under natural conditions, it is able to stop viral spread at the point of entry, making these trees resistant to PPV in the field. ‘Jojo’ has been used as a source for breeding different resistance-generating varieties, such as ‘Jofela’, ‘Moni’ or the interspecific hybrids ‘Docera 6′ (= ‘Dhypres6′, *P. domestica* × *P. cerasifera*) and ‘Dospina 235′ (= ‘Dhprs2′, *P. domestica* × *P. spinosa*), all displaying similar HR. Studies have been conducted in an attempt to identify the genes responsible for this resistance, but the key factors are still unknown [33].

Recently, in an orchard of ‘Jojo’ trees near the city of Herrenberg in Germany, a single tree was found displaying typical PPV symptoms such as leaf curling, chlorosis and fruit damage. The corresponding ‘Jojo’ tree was grafted onto a PPV-sensitive rootstock. The tree was immediately eradicated in order to avoid the escape of a putative resistance-breaking PPV isolate, but some branches were used for controlled propagation of the viral isolate for further study. We report here the use of the propagated infected tissue for amplification by PCR of the consensus viral sequence to clone the new isolate termed PPV-D ‘Herrenberg’, or PPVD-H. This isolate was tested in different resistant plum varieties for its ability to infect avoiding the HR, confirming that in all cases, it could spread in *Prunus* trees, causing standard PPV symptomatology. We also engineered different viral chimeras and identified three amino acids in the NIa region responsible for the evading phenotype. One of those amino acids was involved in proteolytic processing, suggesting cleavage modulation was important to develop the HR-escaping response.

## 2. Results

### 2.1. Genome Sequence Analysis of the HR-Escaping PPV Isolate

**Figure 1:**
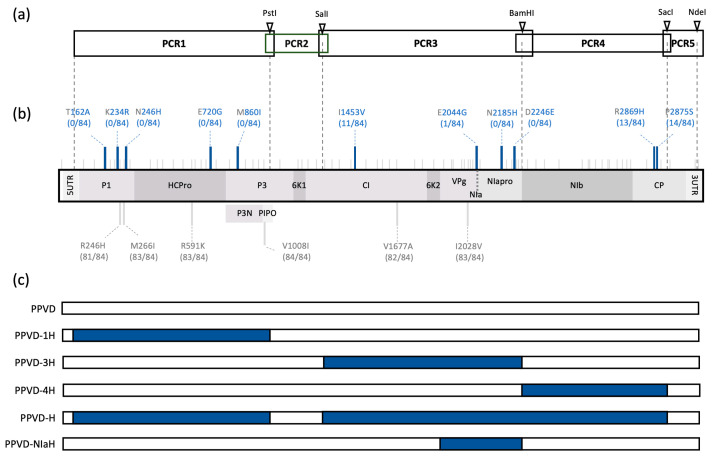
Cloning of PPVD-H and its comparison with other PPV-D isolates. (**a**) Schematic representation of different PCR products amplified for the cloning of PPVD-H. Unique restriction sites present in the PPV sequence are marked with triangles. (**b**) Differences observed between PPVD-H and the standard PPVD isolate. Nucleotide changes are shown on top of the genome as small gray lines; amino acid changes are shown as large lines: common changes are depicted in gray below the genome and rare changes are depicted in blue on top of the genome; amino acid number is indicated, as well as its identity in the two variants PPVD (left) and PPVD-H (right); number of times the amino acid of PPVD-H appears in the 84 PPV-D isolates of SharCo Database is shown in parenthesis. (**c**) Scheme of the viral chimeras generated for characterization of PPVD-H isolate; PPVD and PPVD-H sequences are shown in white and in blue, respectively.

As mentioned earlier, in an orchard in Germany in which ‘Jojo’ trees were used as scions, a tree was found showing typical PPV symptomatology. Initial ELISA tests confirmed it was infected by a PPV virus of the D strain. The tree was rapidly removed and some tissue was kept for further inoculation and study. No ‘Jojo’ trees were available at the time to be used as rootstock, so a susceptible variety, a seedling of *Prunus cerasifera*, was used instead for viral propagation. Infected tissue of this tree was employed to start cloning of the specific isolate. We considered using deep sequencing analysis to identify viral consensus but decided to follow the scheme depicted in Figure 1a. This approach allowed us to read a reliable sequence performing PCR amplifications of cDNA fragments smaller than 3.5 Kb and to generate viral chimeras to define the amino acids relevant for escape of the HR. Each fragment was amplified in two independent PCRs to discern changes in the sequence from possible PCR mistakes. Fragments were cloned into intermediate vectors. We started with the two smallest fragments (PCR2 and PCR5) and compared the obtained sequences with the sequences of a PPV-D isolate available in the laboratory, termed hereinafter PPVD (Accession EF569214) [34]. There were seven nucleotide changes in total, all synonymous, which suggested they were not involved in the observed phenotype. Before we continued, ungrafted, rooted ‘Jojo’ plants were inoculated with *P. cerasifera*-infected tissue to make sure the mutations that facilitated escape from HR were not lost during virus propagation in a susceptible host. Inoculated ‘Jojo’ showed typical PPV symptomatology and we used this tissue to amplify genome regions covered by PCR1, PCR3 and PCR4. Comparison of these regions with PPVD resulted in 76 nucleotide changes, 17 being non-synonymous. We reconstructed in silico the sequence of the isolate, which was termed PPV-D ‘Herrenberg’ or PPVD-H (Accession OQ389521). To evaluate the amino acid variations found in the latter regions sequenced, we aligned sequences from 84 PPV-D isolates from the SharCo Database [35] and compared them with the PPVD-H sequence (File S1). We identified 11 amino acids that were present in less than 15% of the compared sequences, seven of them being unique in the case of PPVD-H. Results are depicted in Figure 1b. We then used the intermediate clones to prepare viral chimeras containing PCR1 (PPVD-1H), PCR3 (PPVD-3H), PCR4 (PPVD-4H) or the three regions at once that would correspond to the newly identified isolate (PPVD-H). Later on, we included another chimera carrying the NIa region of PPVD-H (PPVD-NIaH) (Figure 1c).

### 2.2. PPVD-H Escapes the Hypersensitive Response of Resistant Plum Causing Typical PPV Symptomatology

**Figure 2:**
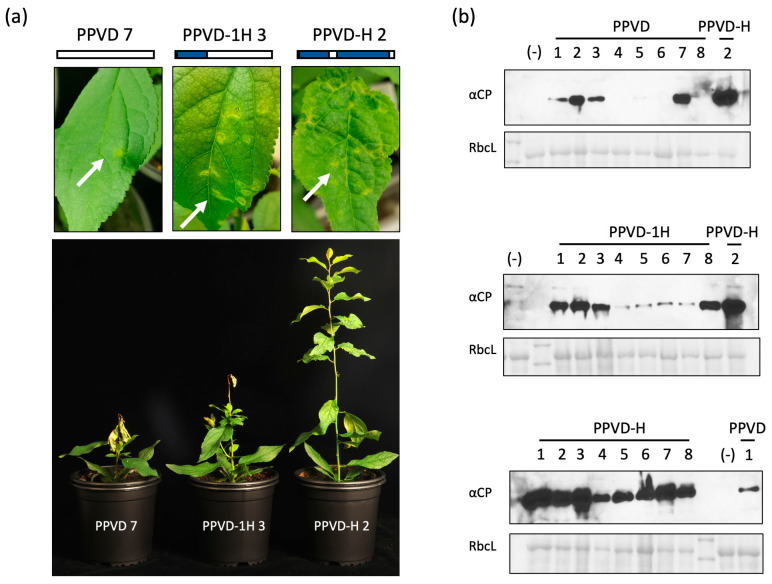
Testing of PPVD, PPVD-1H and PPVD-H in ‘Docera 6′ resistant plants. (**a**) Images were taken at 14 dpi (upper part) from leaves showing different symptoms depending on the inoculated virus; number following the viral name indicates the number of the plant; small scheme of the viral constructs is depicted: PPVD and PPVD-H sequences are shown in white and in blue, respectively; white arrows mark the observed symptoms; the image in the lower part was taken at 25 dpi. (**b**) Anti-CP immunoblots of protein extracts from tissue collected at 21 dpi from upper non-inoculated leaves of the different plants; the numbers on top of each lane correspond to the number of the plant; a sample from PPVD-H or PPVD was loaded in each gel as control; negative control, marked with (−), corresponds to a healthy ‘Docera 6′ plant; a Ponceau red-stained blot (RbcL) is shown below each membrane as loading control.

Identification of the genome sequence of the PPV isolate involved in the HR evasion was followed by testing the corresponding cDNA construct, PPVD-H, in a plant inoculation experiment. ‘Docera 6′ plants were used for biolistic inoculation using PPVD as control and including PPVD-1H to advance in the identification of the possible amino acids responsible for the escaping phenotype. Eight plants were inoculated with each construct and infection was monitored over time. At 14 days post-inoculation (dpi), chlorosis and curling were apparent in upper non-inoculated leaves in most plants (6/8) treated with PPVD-H. In contrast, plants inoculated with PPVD-1H (6/8) presented necrotic spots in upper non-inoculated leaves. PPVD-inoculated plants also presented necrotic spots. In this case, symptoms were observed in half of the plants (4/8) and, at this time of infection, appeared to be milder compared to those of PPVD-1H (Figure 2a, upper panel). At 18 dpi, all plants inoculated with PPVD-H presented typical PPV symptoms in upper leaves. All PPVD-1H-treated plants also presented symptoms, but in this case, they consisted of the appearance of necrotic spots or the spread of the previously detected necrotic foci followed by the fall of the inoculated leaves. Similar results were observed in 5/8 plants inoculated with PPVD with the appearance or spread of necrotic foci on the upper systemic leaves. In addition, plants presenting necrotic spots showed stunting and growth arrest. At 25 dpi, progression of the symptoms in newly born leaves could be observed in plants inoculated with PPVD-H. On the other hand, the apical part of all plants inoculated with PPVD-1H and plants inoculated with PPVD that showed necrotic lesions started to dry and leaves began to fall (Figure 2a, lower panel). This phenotype was similar to the one observed in the grafting experiments with ‘Jojo’ plants and several PPV strains [29,30,31,32]. Three of the plants inoculated with PPVD (plants 4, 6 and 8) remained symptomless throughout the process. Immunoblot analysis of upper leaves was used to correlate the presence of symptoms with the expression in these leaves of the CP protein of PPV (Figure 2b). Western blot analysis of the inoculated leaves confirmed the presence of CP in all plants indicating that virus was inoculated but did not progress systemically in all cases (Figure S1a). RT-PCR analysis of one plant from each construct was used to verify the viral sequences used in each case.

Additionally, five plants inoculated with each construct (1–3, 7, 8 for PPVD; 1–3, 5, 6 for PPVD-1H; 1–5 for PPVD-H) were used for one vernalization cycle. At 10 days post-vernalization (dpv), new leaves started to grow in all plants, and at 20 dpv, symptoms appeared. They were similar to those before, with curling and chlorosis of leaves in PPVD-H-inoculated plants and necrotic lesions in the case of PPVD and PPVD-1H-inoculated plants. Plant 8, inoculated with PPVD, remained asymptomatic throughout the process. At 31 dpv, tissue was collected from three of the plants from each construct, and Western blot analysis confirmed the presence of PPV CP in these leaves (Figure S1b). These results show that PPVD-H is able to systemically infect the resistant plum, displaying a phenotype similar to the one observed in the German orchard ‘Jojo’ tree, compared to the death phenotype caused by PPVD. Moreover, PPVD-1H results show that the 1H region alone is not responsible for the HR escaping phenotype.

### 2.3. The NIa Region of PPVD-H Is Sufficient to Avoid the Hypersensitive Response of Resistant Plum

Once it was confirmed that the PPVD-H isolate avoided HR in ‘Jojo’-based resistant plants, we continued identification of the key regions involved in this escape by testing PPVD-3H and PPVD-4H in ‘Docera 6′ plants. We included a PPVD chimera carrying NIa from PPVD-H (PPVD-NIaH), which is the region presenting unique amino acid changes yet to be tested. We used six plants for each construct. We also used PPVD-H and PPVD as controls, inoculating in this case three resistant (‘Docera 6′) and three susceptible (two ‘Weiwa’ (*Prunus domestica* ‘Wangenheims’) and one ‘Hz’ (*P. domestica* ‘Hauszwetsche’)) plants which had been propagated in vitro.

**Figure 3:**
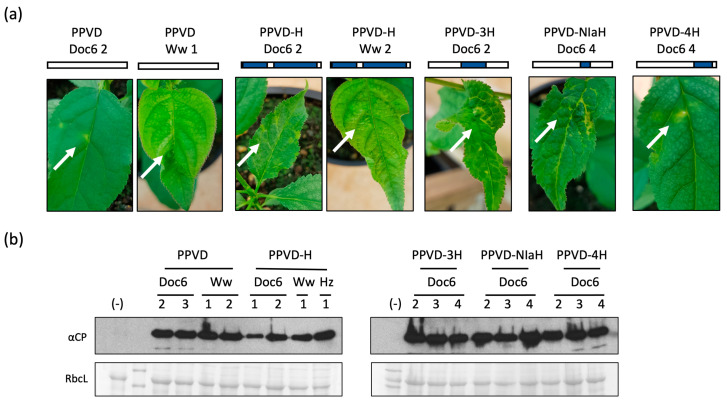
Testing of PPVD-3H, PPVD-NIaH and PPVD-4H in ‘Docera 6′-resistant plants. (**a**) Images taken at 14 dpi from leaves showing different symptoms depending on the inoculated virus; Doc6, Ww and Hz correspond to ‘Docera 6′ (resistant) and ‘Weiwa’ and ‘Hz’ (susceptible) plants, respectively; number following the type of plant indicates the number of the plant; small scheme of the viral constructs is depicted: PPVD and PPVD-H sequences are shown in white and in blue, respectively; white arrows mark the observed symptoms. (**b**) Anti-CP immunoblots of protein extracts from tissue collected at 20 dpi from the upper non-inoculated leaves of the different plants; numbers on top of each lane correspond to the number of the plant; negative control, marked with (−), corresponds to a healthy ‘Docera 6′ plant; a Ponceau red-stained blot (RbcL) is shown below each membrane as loading control.

At 13 dpi, symptoms were already apparent in some of the plants. In the case of the controls, susceptible plants showed mild symptomatology with some chlorosis in upper non-inoculated leaves. This chlorosis was more intense and covered larger parts of the leaf blade in resistant plants inoculated with PPVD-H, while resistant plants inoculated with PPVD developed necrotic spots. General fall of the leaves accompanied by growth arrest and death of the apical part was observed at later times in these plants. The same HR phenotype was observed in ‘Docera 6′ plants inoculated with PPVD-4H, but plants inoculated with PPVD-3H or PPVD-NIaH displayed leaf curling and chlorosis similar to PPVD-H-treated controls (Figure 3a). Western blot analysis confirmed the presence of CP in symptomatic leaves (Figure 3b) and RT-PCR amplification of appropriate cDNA fragments verified the corresponding viral sequences. These results discard the PCR4 region as relevant to avoiding HR and signal NIa with its three amino acid changes as sufficient for developing standard PPV symptomatology.

### 2.4. All Three Amino Acid Changes Found in PPVD-H NIa Are Necessary to Escape the Hypersensitive Response of Resistant Plum

Following identification of NIa as the region responsible for the HR evasion, we intended to evaluate which of these amino acid changes were essential. We engineered single mutants on a PPVD background (PPVD-VPgH, PPVD-NIa1H and PPVD-NIa2H) and a double mutant carrying the two mutations present in NIapro (PPVD-NIa12H). We tested these mutants on a different variety of ‘Jojo’-derived resistant plants, ‘Dospina 235′, using four plants for each construct. We also inoculated in each case two plants of susceptible ‘Weiwa’ to verify that HR was only elicited in the resistant plants. As control, we used in this case PPVD, PPVD-H and PPVD-NIaH.

**Figure 4:**
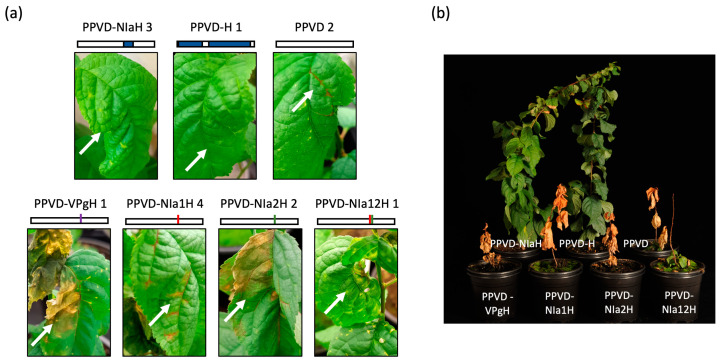
Testing of single and double mutants of PPVD-NIaH in ‘Dospina 235′-resistant and ‘Weiwa’-susceptible plants. (**a**) Images taken at 21 dpi from leaves showing different symptoms depending on the inoculated virus; all images correspond to ‘Dospina 235′ plants; the number following the viral name indicates the number of the plant; small scheme of the viral constructs is depicted: PPVD and PPVD-H sequences are shown in white and in blue, respectively; mutations in VPg, NIa1H and NIa2H are shown in purple, red and green, respectively; white arrows mark the observed symptoms. (**b**) Image of full plants taken at 134 dpi.

At 16 dpi, symptomatology in the case of PPVD-H- and PPVD-NIaH-inoculated resistant plants was similar to before, with chlorosis and curling in the upper non-inoculated leaves. HR in ‘Dospina 235′ plants inoculated with PPVD was a little different from ‘Docera 6′ and instead of necrotic spots, vein necrosis was observed followed by partial necrosis of a large area in the leaf before falling. All single mutants tested behaved similarly to PPVD, indicating that these amino acid changes alone were not enough to escape the HR of the plant. PPVD-NIa12H-inoculated plants displayed a different phenotype early on, somewhat intermediate between PPVD and PPVD-H, starting with curling of the leaves and chlorosis but then turning into necrosis and death of the leaves (Figure 4a). ‘Weiwa’ control plants inoculated with the different constructs showed systemic infection with the same mild symptomatology in all cases without any signs of HR. Additionally, we observed the plants for longer time after viral inoculation and the phenotype at 134 dpi was quite clear with plants inoculated with PPVD-H and PPVD-NIaH still alive and presenting some leaves with symptoms and death of the rest of the plants (Figure 4b).

### 2.5. Cleavage between Vpg and NIapro Is Modulated by the Amino Acid Changes Found in NIa

**Figure 5:**
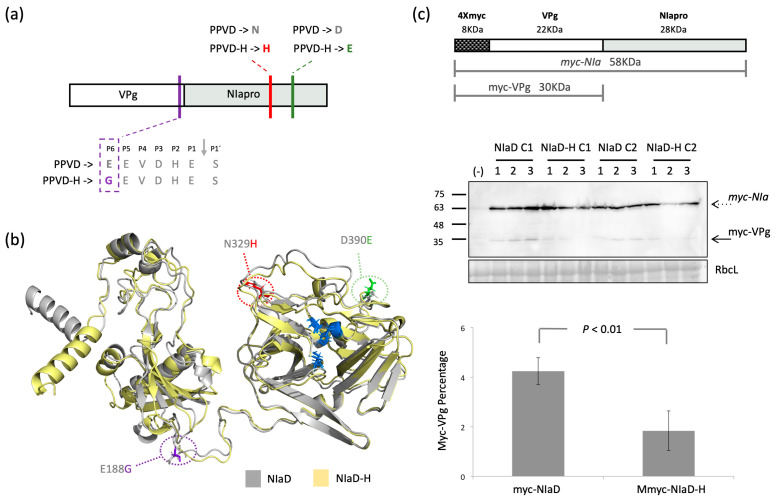
Study of individual mutations in NIa. (**a**) Schematic representation of NIa; mutations in PPVD-VPg, PPVD-NIa1H and PPVD-NIa2H are shown in purple, red and green, respectively; details on the sequence of the proteolytic cleavage site are shown in the case of the VPg change. (**b**) Comparison of the model of NIaD (gray) and NIaD-H (yellow) generated by Alphafold; amino acid changes are marked as sticks following the colors in (**a**); amino acids of the catalytic triad are shown as sticks and colored in blue. (**c**) Transient expression experiment of NIaD and NIaD-H; scheme of the expressed constructs with the corresponding sizes of each product in KDa is shown in the upper part; in the middle part, anti-myc immunoblot of protein extracts from tissue collected at 5 dpi from the different plants; numbers on top of each lane correspond to the number of the plant; C1 and C2 correspond to clone1 and clone 2, respectively; negative control, marked with (−), corresponds to a healthy *N. benthamiana* plant; estimated processed (in Roman text) and unprocessed (in italics) products are marked on the right with solid and dotted arrows, respectively; molecular weight markers are indicated (left; in KDa); a Ponceau red-stained blot (RbcL) is shown below each membrane as loading control; the lower part shows the quantification of the processing ratio from the immunoblot; the difference is statistically significant (*p* < 0.01) by Student´s *t*-test (n = 6).

Proteolytic processing is a tightly regulated mechanism during viral infection [36], involved in host adaptation [37], replication [38] and viral spread [18]. The potyviral endopeptidase NIapro from PPV recognizes a conserved sequence of seven amino acids, [EQN]xVxH[QE]/[STGA], in which ‘x’ is any amino acid, and cleavage occurs between the P1’and P1 positions, indicated as ‘/’ [39]. Changes in any of the conserved positions could have deep effects on cleavage and consequently, on infection. The three amino acid changes between PPVD and the HR-escaping isolate PPVD-H found in NIa are depicted schematically in red, green and purple in Figure 5a and marked with the same colors in the model of PPVD NIa (NIaD) and PPVD-H NIa (NIaD-H) generated with alphafold (Figure 5b) [40]. Amino acid changes in NIapro (Figure 5a,b, marked in red and green) are located at the C-terminal half of the protein and in the model can be seen in the outside region, away from the catalytic triad (Figure 5b, marked in blue). On the other hand, the ‘G’ found in VPg of PPVD-H (Figure 5a,b, marked in purple) is located in the loop between both proteins, as observed in the model, and specifically, in the P6´position of the cleavage site, altering the conserved amino acids [EQN]. This in silico observation suggested that the proteolytic processing of NIa could have been modified. To verify this, we generated different constructs for transient expression in *Nicotiana benthamiana*, pGWB718-NIaD and pGWB718-NIaDH, in which NIaD and NIaD-H, respectively, were fused at the N-terminal part to a myc tag that would allow detection of the uncleaved myc-NIa protein and the corresponding myc-VPg product by Western blot analysis. We used two independent clones from each construct and inoculated three plants per clone. Plants were co-inoculated with tombusviral RNA silencing suppressor P19. Samples were collected at 5 dpi. Immunoblot analysis is shown in Figure 5c, middle panel. Quantification of the percentage of cleaved myc-VPg compared to the uncleaved product (Figure 5c, lower panel) revealed a significant reduction in cleavage in NIaD-H compared to NIaD. This experiment was repeated a second time, obtaining similar significant reduction. A construction using the single mutation present in VPg was also tested, but unfortunately, expression levels were very low, and the cleavage product was undetectable for quantification.

### 2.6. PPVD-H Outcompetes PPVD in Resistant Plants

**Figure 6:**
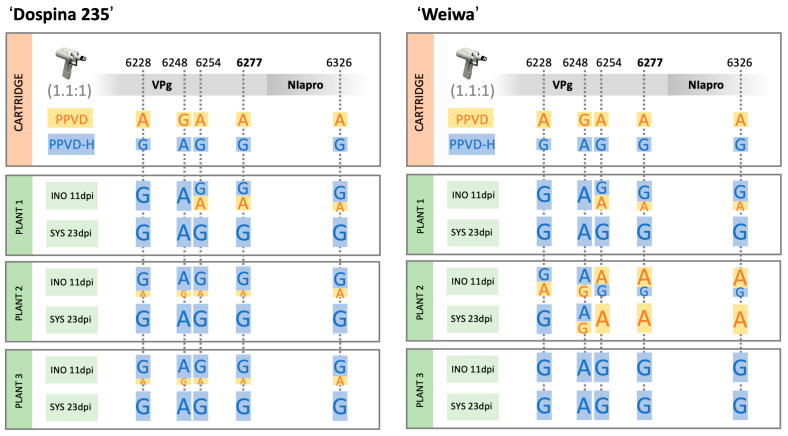
Analysis of the competition experiment between PPVD and PPVD-H in resistant and susceptible plants. Sequencing results with ‘Dospina 235′-resistant plants are shown on the left and results with ‘Weiwa’-susceptible plants are shown on the right; nucleotides in the variable positions are indicated in orange and blue for PPVD and PPVD-H, respectively; the result of the PCR amplification using one of the cartridges as template is shown in the upper part; cartridges were prepared following a PPVD:PPVD-H ratio of 1.1:1; three resistant and three susceptible plants were inoculated; samples for RT-PCR analysis were collected at 11 dpi in the case of the inoculated leaves and at 23 dpi in the case of upper systemic leaves.

Appearance of new viral isolates able to break established resistance is of great concern, and it is important to assess the likelihood of the appearance of these variants and their spread in nature, where competition with other viral species would take place. Answering this question is complicated, but classical competition experiments could help to elucidate the behavior of a newly identified isolate in a more complex scenario. We prepared cartridges mixing PPVD and PPVD-H cDNA in a 1.1:1 ratio and inoculated three resistant ‘Dospina 235′ and three susceptible ‘Weiwa’ plants. We observed symptomatology and collected inoculated leaves at 11 dpi and upper leaves at 23 dpi. We performed RT-PCR from this tissue and amplified a region spanning over part of VPg and NIapro that presented five nucleotide differences between the two viral isolates, including the one resulting in the amino acid change found in VPg. Sequencing of the PCR products allowed us to estimate the ratios of the different variants present in the samples. We also used one of the cartridges as a template for PCR amplification and sequencing to verify how the peaks of the electropherograms reflected the starting viral cDNA input ratios. Results are summarized in Figure 6 and images of the actual electropherograms can be seen in Figure S2. In the case of resistant ‘Dospina 235′ plants, symptoms in the doubly inoculated plants resembled those caused by PPVD-H with chlorosis and curling of the leaves, although growth arrest could also be observed. Sequence analysis was in agreement with this observation. PPVD-H was already imposing on PPVD in all three plants in the inoculated leaves at 11 dpi. In the systemically infected tissue of these plants, collected at 23 dpi, no picks corresponding to PPVD could be observed in any of the five variable positions. The situation in susceptible ‘Weiwa’ plants was different in terms of competition. In one of the plants, plant 3, PPVD-H was the only virus observed in the inoculated or the systemically infected tissue. In another plant, plant 1, PPVD and PPVD-H were present at the inoculated level, but PPVD was absent from the systemically infected leaves. Plant 2, on the other hand, showed greater presence of PPVD in both kinds of leaves. These results suggest that PPVD-H, although more fitted than PPVD, is not a clear dominant species in a non-resistant environment. This is supported by the result obtained with a plant inoculated with PPVD and PPVD-H at a 2:1 ratio in which PPVD was the only viral species detected at 23 dpi.

## 3. Discussion

Plant viruses represent one of the largest threats to agriculture worldwide, enhanced by international trade, population increase and climate change [41]. Control measurements that rely on eradication of the infected material or extensive use of pesticides entail environmental damage and economic losses. The use of resistant crops is an alternative strategy that overcomes these limitations [42]. Breeding programs on European plum were successfully implemented in the past three decades generating several PPV-resistant varieties based on the hypersensitive *Prunus domestica cv.* ‘Jojo’. The appearance of a resistant-breaking isolate poses a threat on the ongoing breeding programs and its characterization is imperative to evaluating the standing risk. At the same time, it offers a great opportunity to study the mechanism of the hypersensitive response to PPV in *Prunus* trees.

The strategy followed for the identification of the new isolate, PPVD-H, was classical overlapping PCR amplification following unique restriction sites for further cloning of the fragments into the PPVD backbone (Figure 1a). Sequencing of each fragment allowed us to build the theoretical sequence of PPVD-H, and comparison with other PPVD isolates showed the amino acids most likely to be responsible for the HR escaping phenotype (Figure 1b). This amplification method in different genome fragments led to the generation of intermediate viral chimeras that were especially useful in further analyses (Figure 1c). Biolistic inoculation of PPVD-H into resistant ‘Docera 6′ plants had already revealed the unique phenotype displayed by this isolate. Resistant plants infected by PPVD showed necrotic lesions that spread over time, growth arrest, fall of the leaves and eventual death. On the other hand, PPVD-H-infected plants presented classical PPV symptomatology with chlorosis and curling of the leaves (Figure 2). Progression of infection in the plant and resurgence of the virus after a vernalization cycle also reproduced PPV behavior in this type of experiment with susceptible plants (Figure S1b). These results validated the obtained viral sequence and gave us a chance to start characterization of this anomalous response. Analyses of the different viral chimeras carrying fragments of PPVD-H over a PPVD background showed that the three amino acid changes present in the NIa region were sufficient to develop an HR escaping phenotype (Figure 2 and Figure 3). Further experiments with single and double mutants confirmed that all three variants were necessary to avoid HR (Figure 4).

Details on how this defensive mechanism works were obtained by studying these amino acid changes at the structural level. VPg change is located at the P6´position of the NIapro cleavage site, an E to G variation that likely affects the proteolytic cleavage of the viral endopeptidase (Figure 5a). This is supported by transient expression experiments carried out *in planta* that showed reduced cleavage of NIaD-H compared to NIaD (Figure 5c). On the other hand, in silico modeling of NIaD and NIaD-H using Alphafold positioned the changes on NIapro in a region that appeared to be independent of this processing site and not involved either with the catalytic triad (Figure 5b). Instead, changes were located in a surface region in flexible loops that could be involved in protein–protein interaction, as was described in the case of *Tobacco etch virus* NIa [43]. These seemingly separated functions suggest a dual mechanism that could be complementary. On the one hand, amino acid mutations in NIapro could be changing the interaction network of this protein or the uncleaved NIa with other viral products or with host factors involved in the response. This, nonetheless, would not be sufficient for the virus to escape the HR, as shown by the intermediate phenotype observed in the case of the virus carrying the NIapro double mutant (PPVD-NIa12H) (Figure 4). Further modulation on the presence of cleaved and uncleaved products appears to be essential and that would be achieved by the additional VPg change in the NIa processing region.

These analyses, however, raise another question: is there a role for the catalytic activity of the protease in the HR beyond the proteolytic processing of NIa? This would imply cleavage of host proteins by the viral endopeptidase and, although an example of this has yet to be reported, a recent work suggests this could be the case [44]. The multiple reports of processing of host proteins by the closely related 3C protease of picornaviruses also support this idea [45]. In addition, there are other examples that follow this hypothesis, such as the bacterial effector AvrPphB that activates the resistance response after cleavage of PBS1 in *Arabidopsis* plants [46,47] or the homologous PVY NIapro that was described as the elicitor of the HR in *Ry*-mediated resistance in potato plants. In this second case, the proteolytic activity was necessary but not sufficient to elicit the response [22,23]. In the case of *Prunus*-resistant trees, the role of PPV NIapro proteolytic activity in the escape of the resistance beyond the processing of NIa is unknown, as it is also undetermined whether the amino acid changes observed in the protease allowed for an active or a passive escape of the HR by altering the interaction network with host proteins. The fact that during viral competition the resistant ‘Dospina 235′ plants did not develop any kind of HR could be indicative of an active mechanism being in place, but this lack of HR could also be explained by the absence of PPVD observed in systemically infected leaves at 23 dpi (Figure 6). Transient expression experiments in ‘Dospina 235′ plants with NIaD and NIaD-H and the corresponding catalytic mutants were unsuccessful and more tests are required in order to clarify this issue.

In terms of risk assessment of this new variant, the competition experiment revealed a clear gain in fitness of PPVD-H over PPVD when they were both tested on resistant *Prunus* genotypes. This superiority was not as apparent when susceptible plants were employed since the presence of PPVD in systemic tissue at 23 dpi was clear in one of the plants (Figure 6). This suggests that the introduced changes are more efficient in the resistant background than in the susceptible environment, and this could be related to the HR escape or to other specific host interactions. In fact, results obtained in resistant plants inoculated with a PPVD-1H chimera (Figure 2), which presented faster development of symptoms than plants inoculated with PPVD, suggest that mutations in P1, HCPro or P3 could be involved in the enhanced adaptation of PPVD-H. The appearance of PPV isolates able to break resistance, such as PPVD-H, is linked to the use of susceptible rootstocks that can become infected by aphid inoculation, generating a large number of viruses that in turn come into contact with the hypersensitive scion cultivar. The use of resistant rootstocks such as ‘Docera 6′ or ‘Dospina 235′ is an important improvement in terms of viral control as it reduces the possibility for selection of resistant-breaking isolates significantly. In that respect, resistant scion cultivars must only be grafted onto resistant rootstocks. For susceptible scion cultivars, susceptible rootstocks have to be used.

Studying PPVD-H allowed us to better understand the mechanism of resistance in these plants. Our experiments indicate that NIa could be the pathogenicity determinant in this case, as was found in other plant–virus pathosystems. They also showed that proteolytic processing of NIa is likely involved in the response, altering the amounts of cleaved and uncleaved products during viral infection. Further studies are needed to elucidate the details of the hypersensitive response, to make it more efficient and to implement its transfer to other susceptible species.

## 4. Materials and Methods

### 4.1. Cloning of PPVD-H and Its Variants

Tissue from *P. cerasifera* and *P. domestica* cv. ‘Jojo’-infected plants was smashed under liquid nitrogen and used for total RNA extraction with a Plant Total RNA purification mini kit for woody plants (Favorgen). cDNA was prepared using Superscript III following manufacturer´s instructions (Thermo Fisher Scientific, Waltham, MA, USA). The actual cloning of the different viral constructs is described in detail in File S2. Briefly, PCR amplifications corresponding to the different fragments were cloned into intermediate plasmids using standard cut-and-paste ligations. From these plasmids, inserts were extracted using restriction enzymes and ligated into a plasmid carrying PPVD background, digested with compatible enzymes. Viral constructs carrying different point mutations were obtained by digestion and ligation of overlapping PCRs performed with primers carrying the corresponding mutations.

### 4.2. Plasmids Used for Transient Expression and Agroinfiltration

Plasmids pGWB718-NIaD and pGWB718-NIaH were prepared using the Gateway system. Initial PCR was performed for amplification of NIaD and NIaD-H using primers 1563/1564, with templates PPVD and PPD-H, respectively. PCR fragments were recombined by BP reaction with pDONR207 (Invitrogen). LR recombination of these plasmids followed, using pGWB718 as destination vector [48]. Plasmid pBIN61:p19 was kindly provided by David Baulcombe (University of Cambridge, UK). For agroinfiltration, *N. benthamiana* plants were grown in a greenhouse with a 16 h light/8 h dark photoperiod maintained at a temperature range of 19 to 23 °C. Plants with 4–5 leaves were infiltrated as described [49], with *A. tumefaciens* strain C58C1-313 [50] carrying the indicated binary plasmid and using an OD_600_ of 0.5 for each construct. Two independent clones for each construct were used to infiltrate three plants. Samples were collected at 5 dpa.

### 4.3. Biolistic Inoculation and RT-PCR Analysis

Prunus plants were grown in a chamber with a 16 h light/8 h dark photoperiod maintained at a temperature of 22 °C. The Helios Gene Gun system (Bio-Rad) was used for biolistic inoculation. Microcarrier cartridges were prepared with 1.0 µm gold particles coated with the different plasmids at a DNA loading ratio of 2 µg/mg of gold and a microcarrier loading of 0.5 mg per shot. Helium pressure was set at 10 bars. Each cartridge was shot twice onto two different leaves of each plant. For RT-PCR analyses, samples were collected at the indicated times and ground in a mortar under liquid nitrogen. Total RNA was extracted with Plant Total RNA purification mini kit for woody plants (Favorgen), and cDNA was prepared using Superscript III following manufacturer´s instructions (Thermo Fisher Scientific). PCR amplifications were performed with primers listed in Table S1 of File S2.

### 4.4. Western Blot Analysis

Plant tissue ground to fine powder under liquid nitrogen was used to prepare protein extracts by homogenization in extraction buffer (125 mM Tris-HCl, 2% SDS, 6 M urea, 5% β-mercaptoethanol, 10% glycerol, 0.05% bromophenol blue, pH 7.5) using 4 mL/g of tissue. Proteins were resolved by electrophoresis on sodium dodecyl sulphate polyacrylamide gels (12% acrylamide) and electroblotted onto nitrocellulose membranes. Anti-PPV CP serum and horseradish-peroxidase-conjugated goat anti-rabbit IgG (Jackson) were used as primary and secondary antibody, respectively, for protein detection. Immunostained proteins were visualized by enhanced chemiluminescence detection with Clarity Western ECL Substrate (BioRad, Hercules, CA, USA).

### 4.5. In Silico Study of PPV-D Alignment and NIa Structure

Eighty-four amino acid sequences from different PPV-D isolates were collected from the SharCo database and used for alignment using ClustalW in Megalign (DNA Star). NIaD and NIaD-H models were obtained using AlphaFold Colab [40].

## Supplementary Materials

The following supporting information can be downloaded at: https://www.mdpi.com/article/10.3390/plants12081609/s1, File S1: PPV alignment; Figure S1: In depth analysis of the infection pattern of PPVD, PPVD-1H and PPVD-H before and after vernalization; Figure S2: Eletropherograms corresponding to RT-PCR amplification and sequencing of samples from the competition experiment between PPVD and PPVD-H; File S2: Materials and Methods.
