# Supplementary material for: The Mechanism of Resistance of EUROPEAN Plum to Plum pox virus Mediated by Hypersensitive Response Is Linked to VIRAL NIa and Its Protease Activity
Source: Plants (Basel). 2023 Apr 10;12(8):1609. doi: 10.3390/plants12081609 (PMC10147044; doi:10.3390/plants12081609)
Supplement: Supplementary file 1 [file plants-12-01609-s001.zip › FileS2.docx]

**File S2**

**Materials and methods**

*Cloning of PPVD-H and its variants*

Tissue from *P. cerasifera* and *P. domestica* cv ‘Jojo’ infected plants was smashed under liquid nitrogen and used for total RNA extraction with Plant Total RNA purification mini kit for woody plants (Favorgen). cDNA was prepared using Superscript III following manufacturer´s instructions (Thermo Fisher Scientific). PCR reactions 1-5 were done using primers listed in Supplementary Table 1. Primers were purchased to Merck. Two independent PCR reactions were done in each case with Phusion High-Fidelity DNA Polymerase (New England BioLabs). Intermediate plasmids and PCR reactions were digested as follows: PCR1 with PstI and p35SeNOSb with PstI/Eco147I, PCR2 and pUC19 with PstI/SalI, PCR3 and pUC19 with BamHI/SalI, PCR4 and pUC19 with BamHI/SacI and PCR5 with SacI and pUC19 with SacI/SmaI. Restriction enzymes were purchased to New England BioLabs. Fragments were ligated into the corresponding plasmids using T4DNA ligase (Thermo Fisher Scientific). Sequencing of all products was carried out by Macrogen. Once the intermediate clones were confirmed, viral chimeras were prepared. *PPVD-1H* was done by digesting PPVD [34] and p35SeNOSb-PCR1 with PstI/PvuII; *PPVD-3H* was done by digesting PPVD and pUC19-PCR3 with BamHI/SalI; *PPVD-4H* was done by digesting PPVD and pUC19-PCR4 with BamHI/SacI. In all cases, PPVD was treated with rSAP (New England Biolabs) before ligation with T4 DNA ligase (Thermo Fisher Scientific). To generate *PPVD-H*, we first generated a plasmid with PCR1 and PCR3 fragments of PPVD-H, PPVD-1H3H, by using PPVD-1H as vector and PPVD-3H as insert and digesting both with SalI/BamHI followed by T4 DNA ligation. We then used that plasmid as vector and PPVD-4H as insert after digestion with BamHI/SacI followed by T4 DNA ligation. To generate *PPVD-NIaH*, two PCR reactions were performed, one using primers 488/3274 and PPVD as template, and another one using primers 3273/266 and PPVD-H as template. Overlapping PCR was performed using primers 488/266 and previous PCR products as templates. As before, PPVD-NIaH was cloned by digestion of the overlapping PCR product and PPVD with enzymes BamHI/SalI followed by ligation. Individual mutants *PPVD-VPgH*, *PPVD-NIa1H* and *PPVD-NIa2H* were prepared as follows: first, 2 PCR reactions in each case were done using PPVD as template and using as primers 488/3323 and 3322/266 for VPg, 488/3325 and 3324/266 for NIa1H and 488/3327 and 3326/266 for NIa2H. Corresponding PCR products were used as templates in overlapping PCRs using primers 488/266. Fragments were digested with BamHI/SalI and cloned into PPVD digested with the same enzymes. To obtain double mutant *PPVD-NIa12H*, two PCR reactions were done: one using as template PPVD-NIa1H and primers 488/3327 and another using as template PPVD and primers 3326/266. Overlapping PCR using primers 488/266 was then performed. PCR fragment was digested with BamHI/SalI and cloned into PPVD digested with the same enzymes.

**Table S1. Primers used in this study**

| **Primer** | **Sequence 5´-> 3´** | **Use** |
| --- | --- | --- |
| 29 | AAAATATAAAAACTCAACAC | PCR1 amplification |
| 441 | TCCTGCAGATAACTTTTTTCAACC | PCR1 amplification |
| 280 | ATCTGCAGGAATTGGAGCAAGC | PCR2 amplification |
| 210 | GGGACAGTTGGTGCAAC | PCR2 amplification; RT-PCR amplification to verify PPVD, PPVD-1H and PPVD-H |
| 488 | CTCAATAAACTCAAAGGC | PCR3 amplification |
| 266 | CCAACTGCTGCTTTCATG | PCR3 amplification |
| 67 | GGATGAAGTTTGCTGG | PCR4 amplification |
| 55 | CTATGCACCAAACC | PCR4 amplification |
| 81 | GAACTTTTACAGTGCCAC | PCR5 amplification |
| 522 | TTTTTTTTTTGTCTCTTGC | PCR5 amplification |
| 266 | CCAACTGCTGCTTTCATG | Cloning of PPVD-NIaH, PPVD-VPgH, PPVD-NIa1H, PPVD-NIa2H, PPVD-NIa12H; RT-PCR amplification and sequencing to verify PPVD-3H, PPVD-NIaH and PPVD-4H, PPVD-VPgH, PPVD-NIa1H, PPVD-NIa2H, PPVD-NIa12H; competition experiment |
| 488 | CTCAATAAACTCAAAGGC | Cloning of PPVD-NIaH, PPVD-VPgH, PPVD-NIa1H, PPVD-NIa2H, PPVD-NIa12H |
| 3273 | CCAAGGCTTCAATCGTAGGC | Cloning of PPVD-NIaH; RT-PCR amplification and sequencing to verify PPVD-3H, PPVD-NIaH and PPVD-4H, PPVD-VPgH, PPVD-NIa1H, PPVD-NIa2H, PPVD-NIa12H; competition experiment |
| 3274 | GCCTACGATTGAAGCCTTGG | Cloning of PPVD-NIaH |
| 3322 | GAGGGGGACGGAGAAGTTGAC | Cloning of PPVD-VPgH |
| 3323 | GTCAACTTCTCCGTCCCCCTC | Cloning of PPVD-VPgH |
| 3324 | CCAGTTGATCACAGTCATTTC | Cloning of PPVD-NIa1H |
| 3325 | GAAATGACTGTGATCAACTGG | Cloning of PPVD-NIa1H |
| 3326 | CAAGACAATGAGAACTGGATAAAG | Cloning of PPVD-NIa2H, PPVD-NIa12H |
| 3327 | CTTTATCCAGTTCTCATTGTCTTG | Cloning of PPVD-NIa2H, PPVD-NIa12H |
| 1563 | GGGGACAAGTTTGTACAAAAAAGCAGGCTCCAT  GGCAGGCTTCAATCGTAGGCAAAGAC | Cloning pGWB718-NIaD/ pGWB718-NIaD-H |
| 1564 | GGGGACCACTTTGTACAAGAAAGCTGGGTTACT  GAGTGTAAACAAATTCCCC | Cloning pGWB718-NIaD/ pGWB718-NIaD-H |
| 3233 | CATGCAGCAGTTCTATGTG | RT-PCR amplification to verify PPVD, PPVD-1H and PPVD-H |
| 264 | TAGTCCGACGGAAAAGAG | Sequencing to verify PPVD, PPVD-1H and PPVD-H |
| 271 | TGTCGTACCTGCCTCC | Sequencing to verify PPVD, PPVD-1H and PPVD-H |

**Figure S1. In depth analysis of the infection pattern of PPVD, PPVD-1H and PPVD-H before and after vernalization.** (a) Anti-CP immunoblots of protein extracts from tissue collected from the first inoculated leaf (left) or the second inoculated leaf (right); numbers on top of each lane correspond to the number of the plant; negative control, marked with (-), corresponds to a healthy ‘Docera 6’ plant; positive control, marked with (+), correspond to a *P. domestica* ‘Brompton’ plant previously inoculated with PPVD; a Ponceau red-stained blot (RbcL) is shown below each membrane as loading control. (b) Images taken at 33 dpv; number following the viral name indicates the number of the plant; anti-CP immunoblot of protein extracts from tissue collected at 33 dpv from three different plants from each construct; numbers on top of each lane correspond to the number of the plant; a Ponceau red-stained blot (RbcL) is shown below each membrane as loading control.

**Figure S2. Eletropherograms corresponding to RT-PCR amplification and sequencing of samples from the competition experiment between PPVD and PPVD-H.** Sequencing results with ‘Dospina 235’ resistant plants are shown on the left and results with ‘Weiwa’ susceptible plants are shown on the right; nucleotides in the variable positions are indicated with black rectangles; result of the PCR amplification using one of the cartridges as template is shown in the upper part; results from the last nucleotide variation 6326 were not included.
