# Supplementary figures and images for: The Mechanism of Resistance of EUROPEAN Plum to Plum pox virus Mediated by Hypersensitive Response Is Linked to VIRAL NIa and Its Protease Activity
Source: Plants (Basel). 2023 Apr 10;12(8):1609. doi: 10.3390/plants12081609 (PMC10147044; doi:10.3390/plants12081609)

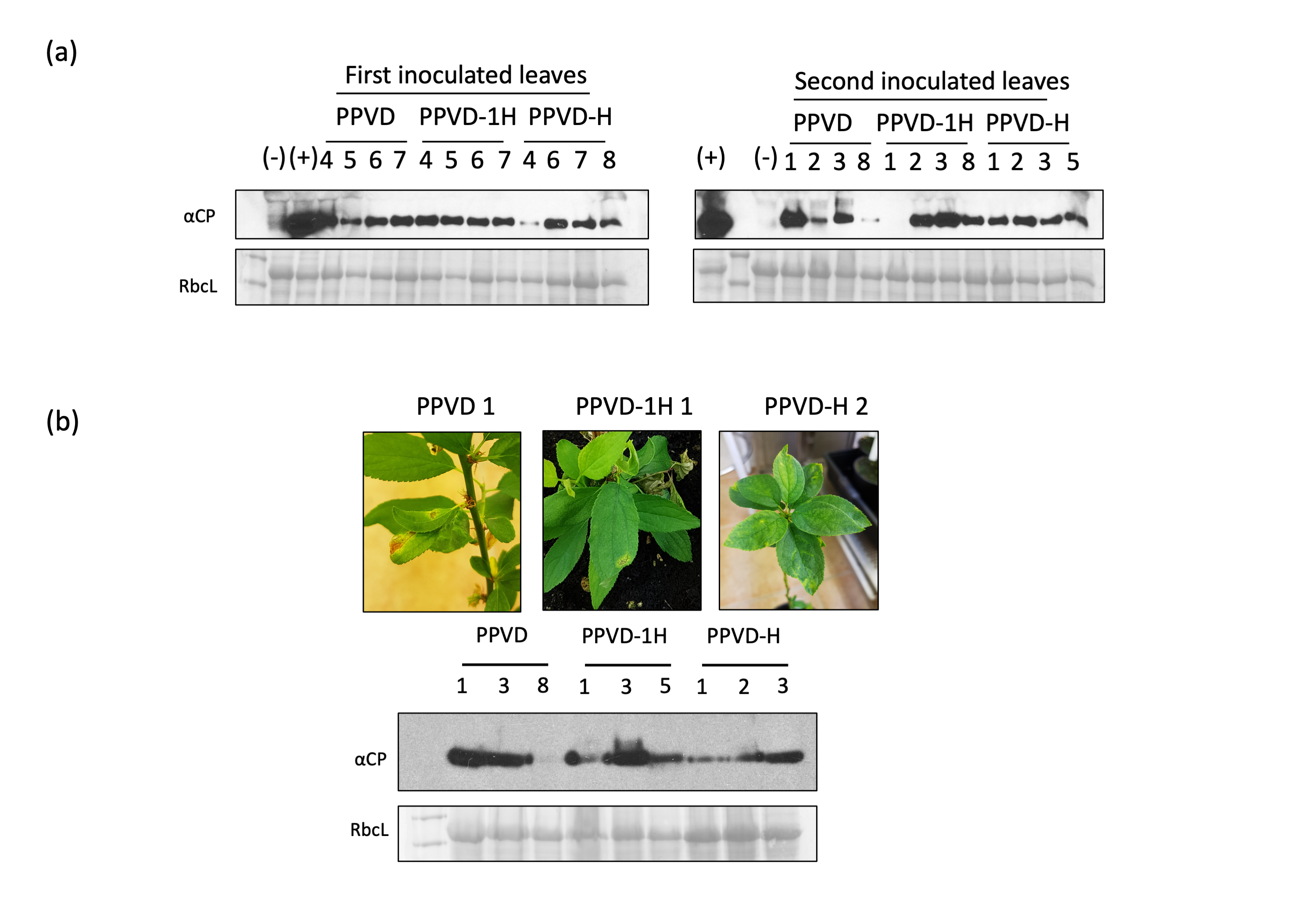

Supplement: Supplementary file 1 [file plants-12-01609-s001.zip › FigureS1.tiff]

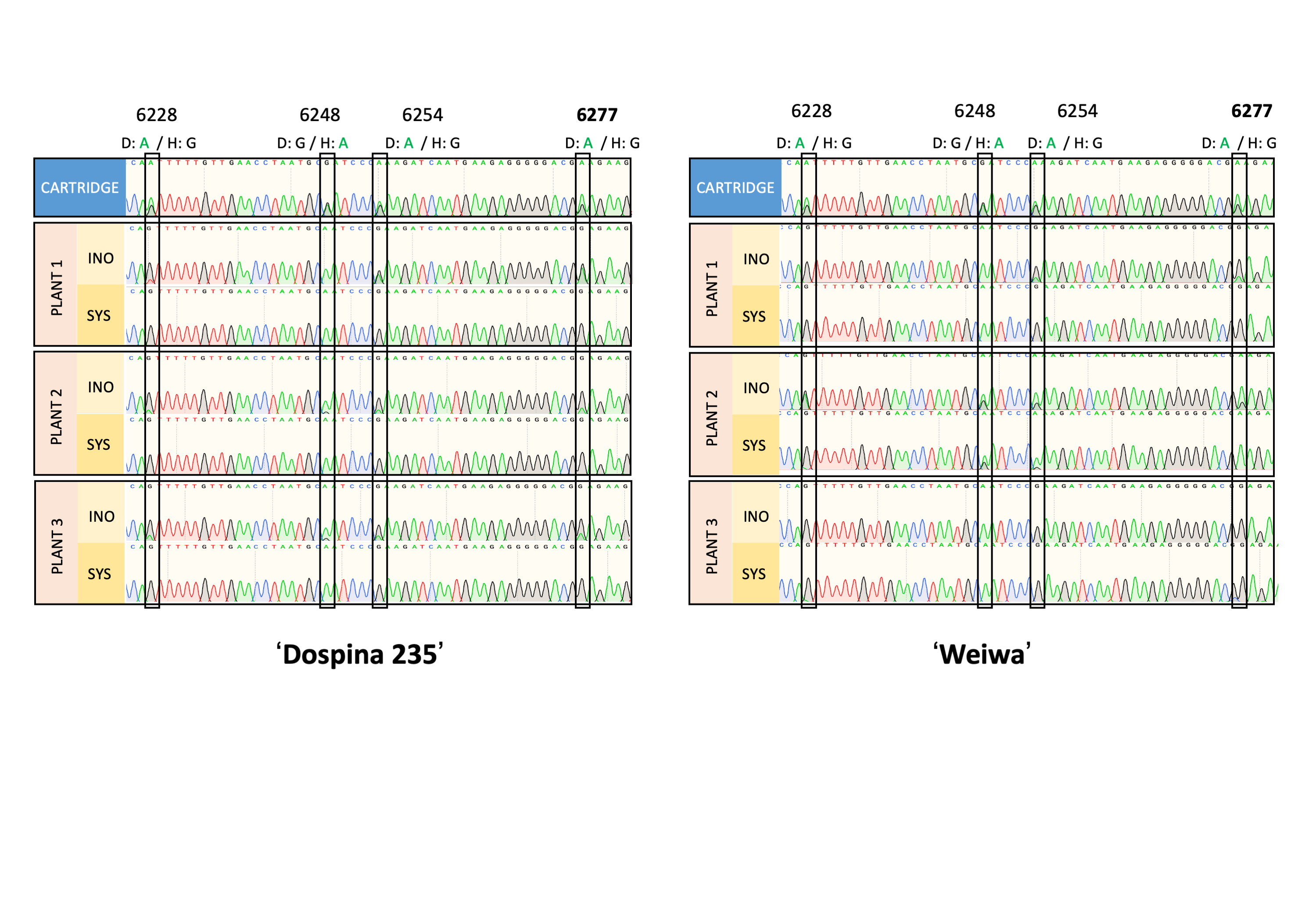

Supplement: Supplementary file 1 [file plants-12-01609-s001.zip › FigureS2.tiff]
